# Anti-Tumor Effects of *BDH1* in Acute Myeloid Leukemia

**DOI:** 10.3389/fonc.2021.694594

**Published:** 2021-06-04

**Authors:** Fei Han, Huanhuan Zhao, Jun Lu, Weina Yun, Lingling Yang, Yude Lou, Dan Su, Xin Chen, Shixuan Zhang, Hanwei Jin, Xiang Li, Jie Sun, He Huang, Qishan Wang, Xi Jiang

**Affiliations:** ^1^ Department of Pharmacology and Bone Marrow Transplantation Center of the First Affiliated Hospital, Zhejiang University School of Medicine, Hangzhou, China; ^2^ Institute of Hematology, Zhejiang University & Zhejiang Engineering Laboratory for Stem Cell and Immunotherapy, Hangzhou, China; ^3^ Liangzhu Laboratory, Zhejiang University Medical Center, Hangzhou, China; ^4^ Bone Marrow Transplantation Center of the First Affiliated Hospital, Zhejiang University School of Medicine, Hangzhou, China; ^5^ College of Animal Sciences, Zhejiang University, Hangzhou, China

**Keywords:** *BDH1*, ketone, metabolism, AML - acute myeloid leukemia, tumor suppressor, prognostic factors

## Abstract

Dysregulation of ketone metabolism has been reported in various types of cancer. In order to find out its role in acute myeloid leukemia (AML) pathogenesis, we first analyzed the expression levels of 10 key genes involved in ketone metabolism in AML blasts and CD34^+^ hematopoietic stem cells (HSCs) from healthy donors. We found that the expression level of *BDH1* was significantly lower in AML than in normal HSCs. The downregulation of *BDH1* gene expression in AML cell lines as compared with normal HSCs was further confirmed with real-time RT-PCR. Analysis of TCGA and other database revealed that the downregulation of *BDH1* was associated with worse prognosis in AML patients. In addition, we showed that overexpression of *BDH1* inhibited the viability and proliferation of AML cells. In contrast, *BDH1* knock-down promoted AML cell growth. Collectively, our results suggest the previously unappreciated anti-tumor role of *BDH1* in AML, and low *BDH1* expression predicts poor survival.

## Introduction

Acute myeloid leukemia (AML), the most common type of acute leukemia in adults, is a group of highly aggressive heterogeneous cancer (1). Despite of intensive traditional chemotherapy, the overall 3-year survival of AML patients was only around 25%. Many subtypes of AML are associated with intermediate to poor prognosis, especially in elder patients (2). Therefore, it is urgently needed to better understand the molecular mechanism of AML, and develop novel therapy targeting key molecules crucial for leukemogenesis.

Energy reprogramming is a hallmark in cancer. Dysregulation of metabolism, including the aerobic glycolysis (3–5), the amino acid (6) and fatty acid metabolism (7, 8), has been found in cancer, including AML. Targeting metabolic pathways provides a potential strategy for cancer therapy (9). Among the metabolic pathways, ketone metabolism, which includes hepatic ketogenesis and extrahepatic ketolysis, was used to be recognized as the byproduct of lipid metabolism. A bunch of regulators, such as the 3-hydroxymethylglutaryl-CoA synthase (HMGCS2), the hydroxymethylglutaryl coenzyme A lyase (HMGCL), the 3-oxoacid CoA transferase 1 (OXCT1), and the phosphatidylcholine-dependent mitochondrial βOHB dehydrogenase (BDH1), etc., are involved in the process. During ketogenesis, the condensation of AcAc-CoA and acetyl-CoA was catalyzed by HMGCS2 to generate hydroxymethylglutaryl (HMG)-CoA. HMGCL cleaves HMG-CoA to release acetyl-CoA and AcAc. AcAc is then converted into βOHB by BDH1 in a NAD^+^/NADH^-^ coupled near-equilibrium reaction. During ketolysis, extrahepatic mitochondrial BDH1 catalyzes the conversion between βOHB and AcAc, and OXCT1 mediates the conversion of AcAc to AcAc-CoA. A reversible AcAc-CoA thiolase reaction catalyzed by any of the four mitochondrial thiolases ACAT1, ACAT2, HADHA, HADHB yields acetyl-CoA, which thereafter enters the TCA cycle for ATP production (10).

Several clinical and preclinical studies suggest the anti-tumor effect of ketone diet (KD) in solid tumors (11), such as glioma (12) and pancreatic cancer (13). It is also shown that KD can improve the response of PI3K inhibitor BKM120 in *MLL-AF9* AML mouse model (14). However, the effects of KD in cancer patients still remain quite controversial (15). Up till now, knowledge about the ketone metabolism in AML is very limited. Therefore, it is necessary to identify the role and molecular mechanism of ketone body metabolism in AML.

In this study, we show the downregulation of key genes involved in ketone body metabolism in AML blasts as compared with normal hematopoietic stem cells (HSCs), and identify the association between *BDH1* expression and AML prognosis. The anti-tumor role of *BDH1* was then further confirmed in AML cells. Together, we show the anti-tumor effects of *BDH1* in AML, which indicate the therapeutic potential of targeting *BDH1* to cure AML.

## Materials and Methods

### Plasmid Construction

The CDS of *BDH1*, encoding the human *BDH1* gene, was amplified through PCR using primers 5′-TATAGCTAGCATGCTGGCCACCCG-3′ and 5′-TATAACCGGTTCAGCGGATGTAGATCAT-3′, and then cloned into pLJM1-EGFP lentiviral vector (Addgene, 19319). The CDS of mouse *Bdh1* gene was amplified through PCR using primers 5′-TATACTCGAGATGCTAGCTGCC-3′ and 5′- TATAGAATTCTCAGTGTATGTAGATCTTGT-3’, and then ligated into a retroviral vector, namely MSCV-PIG (Addgene, 105594).

### Cell Culture

THP1 cells were maintained in RPMI 1640 supplemented with 10% FBS, and 1% penicillin-streptomycin. Mouse leukemic cells were kept in RPMI 1640 supplemented with 10 ng/mL interleukin 3 (IL-3), 10 ng/mL IL-6, 100 ng/mL SCF, 10% FBS and 1% penicillin-streptomycin. Human HSCs were maintained in IMDM supplemented with 20% FBS, 1% penicillin-streptomycin, 10 ng/mL IL-3, 10 ng/mL IL-6, 10 ng/mL Flt3 ligand, 10 ng/mL TPO, and 100 ng/mL SCF.

### Lentivirus Production and Infection

pMD2.G, psPAX2, pLJM1-EGFP plasmids were purchased from Addgene (Cambridge, MA). 0.7 μg pMD2.G, 0.7 μg psPAX2 and 1.5 μg pLJM1-EGFP constructs, i.e. pLJM1-BDH1 or pLJM1-EGFP control were co-transfected into HEK-293T cells in a 60 mm cell culture dish with Lipofectamine 2000 reagent (ThermoFisher, 11668). Lentiviral particles were harvested 48 and 72 hours after transfection. The lentivirus particles were directly added into leukemic cells and two rounds of ‘spinoculation’ (16–18) were performed to allow the infection of viruses.

### Retrovirus Production and Infection

Retrovirus vectors were co-transfected with pCL-Eco packaging vector into HEK293T cells using Lipofectamine 2000 reagent (ThermoFisher, 11668) to produce retrovirus. BM cells were harvested from 6-week-old B6.SJL (CD45.2) mice after five days of 5-fluorouracil (5-FU) treatment, and primitive hematopoietic progenitor cells were enriched with Mouse Lineage Cell Depletion Kit (Miltenyi Biotec Inc., 130-090-858). An aliquot of enriched hematopoietic progenitor cells was added to retroviral supernatant together with polybrene in cell culture plates, which were centrifuged at 2,000 g for 3 hours at 30°C [i.e., ‘spinoculation’ (16–18)] and then the medium was replaced with fresh medium and incubated for 20 hours at 37°C. Next day, the same procedure was repeated once. Cultures were incubated at 37°C in a humidified atmosphere of 5% CO_2_ in air.

### Cell Proliferation Assays

10,000 cells were seeded into each well of a 96-well plate, and allowed to grow for 4-5 days to build a growth curve. For cell viability assay, cells were seeded into 96-well plates in triplicates and MTT (Sigma, M2128) was used to assess cell proliferation and viability following the manufacturer’s instructions. Briefly, cells were seeded at a density of 5000-10000 cells/100 μL. Dye solution was added at indicated time points and incubated at 37°C for 3-4 hours before adding of solubilization/stop solution to stop the reaction. The absorbance at 540 nm was read at the end point using SynergyMx M5.

### Quantitative Real-Time PCR Analysis

For quantitative real-time PCR (qRT-PCR) analysis, the cDNAs were amplified using HiScript^®^ II Q RT SuperMix (Vazyme, R223) for qRT-PCR. Relative gene expression levels were detected through qRT-PCR using the ChamQ™ SYBR^®^ qPCR Master Mix (Vazyme, Q311) and LightCycler^®^ 480 System. Using GAPDH as an internal reference, the ΔΔCT method was used to quantify the relative expression of target genes. The primer sequences were as follows: *GAPDH*-Forward, 5’-AATCCCATCACCATCTTCCAG-3’; *GAPDH*-Reverse, 5’-AAATGAGCCCCAGCCTTC-3’; *BDH1*-Forward, 5’-GGCAGAAGTGAACCTTTGGG-3’; *BDH1*-Reverse, 5’-GCAGTCCGAGAAAGCCTCTAC-3’; *Gapdh*-Forward, 5’-AGGTCGGTGTGAACGGATTTG-3’; *Gapdh*-Reverse, 5’-TGTAGACCATGTAGTTGAGGTCA-3’; *Bdh1*-Forward, 5’-TTCCCCTTCTCCGAAGAGC-3’; *Bdh1*-Reverse, 5’ -CCCAGAGGGTGCATCTCATAG-3’.

### Data Set Processing

The RNA-seq databases (TCGA-LAML, n=151) and patient information were obtained from National Cancer Institute GDC Data Portal. The RNA-seq and patient clinical data in BEATAML database were downloaded from cBioPortal online tool (https://www.cbioportal.org). The Agilent microarray data sets (GSE9476, GSE6891, GSE24006, GSE65409) were obtained from Gene Expression Omnibus (GEO) database.

The Agilent microarray probes IDs for GSE9476, GSE6891, GSE24006 and GSE65409 were annotated using the platform GPL96 [(HG-U133A) Affymetrix Human Genome U133A Array], GPL570 [(HG-U133_Plus_2) Affymetrix Human Genome U133 Plus 2.0 Array] and GPL10881 [Affymetrix Human Genome U133 Plus 2.0 Array (CDF: HGU133Plus2_Hs_REFSEQ_12.1.0)], GPL6947 (Illumina HumanHT-12 V3.0 expression beadchip), respectively. When multiple probes mapped to the same gene, the mean of the signal intensities was used.

### Survival Analysis

To investigate the association of gene expression with survival time in the TCGA-LAML and GSE6891 database, the median expression level was employed to divide the patients into expression-high and expression-low group. Kaplan-Meier plot and log-rank (Mantel-Cox) test were used to compare overall survival months between two groups.

### Unsupervised Hierarchical Clustering Analysis

Unsupervised hierarchical clustering of 8 ketone metabolism genes was conducted using the Pheatmap function in R package pheatmap. cDNA microarray expression data were subjected as a numeric matrix to clustering analysis, using ‘complete linkage’ as clustering method, using ‘Euclidean Distance’ as distance method.

### Statistical Analysis

Statistical analysis was performed by using GraphPad Prism version 8.0 (GraphPad Software, San Diego, CA). The independent Student’s *t*-test was used to analyze the results and data are expressed as the means ± SEM. Kaplan-Meier plots were analyzed for survival analysis. The Mann-Whitney test was used for non-normally distributed data and was analyzed by IBM SPSS Statistics 21 software for Windows (SPSS Inc., Chicago, IL, USA).

## Results

### Expression of *BDH1*, *HADHB*, and *OXCT1* Is Down-Regulated in AML

**Figure 1:**
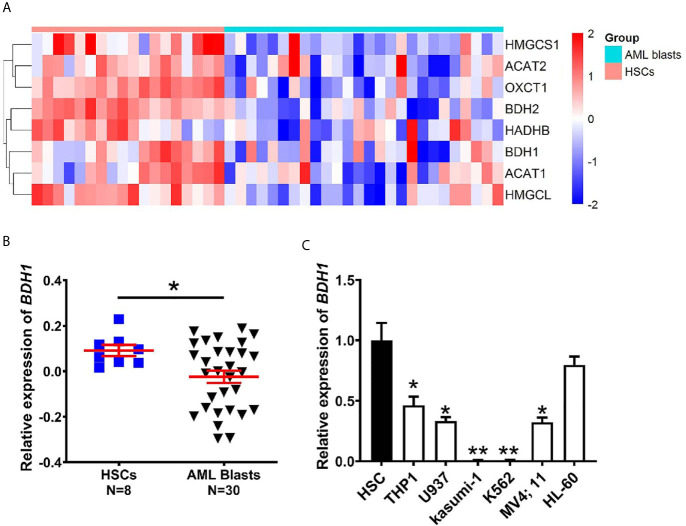
The expression of *BDH1* is downregulated in AML as compared with normal HSCs. **(A)** A heatmap of the hierarchical clustering of 8 key genes involved in ketone body metabolism. Input data are the mRNA expression values of AML blasts over normal HSCs. Upregulated genes are designated with red color, while blue color indicates downregulated genes. **(B)** The mRNA expression analysis of *BDH1* in 30 cases of AML blasts and 8 cases of normal HSCs in GSE65409 database. The expression values in GSE65409 were log2-transformed and mean centered. The *P* values were detected by *t*-test. **(C)** The expression level of *BDH1* was determined by qRT-PCR in AML cell lines and normal HSCs, normalized to *GAPDH*. *p < 0.05, **p < 0.01.

To explore the expression pattern of genes participating in ketogenesis and ketolysis in AML, a total of 10 genes from “Synthesis and degradation of ketone bodies” (hsa00072) of Kyoto Encyclopedia of Genes and Genomes (KEGG) were selected for the following analysis (**Table 1**). The 10 genes were: *ACAT1*, *ACAT2*, *BDH1*, *BDH2*, *HMGCS1*, *HMGCS2*, *HMGCL*, *HMGCLL1*, *OXCT1*, *OXCT2*. We analyzed the cDNA microarray data of 26 cases of AML blasts and 18 cases of normal HSCs from GEO database (GSE9476), and found information of 9 of the above 10 genes (except *HMGCLL1*, which was not included in the database) from the database. Results show that expression levels of 8 of the 9 genes, i.e., *ACAT1*, *ACAT2*, *BDH1*, *BDH2*, *HADHB*, *HMGCL*, *HMGCS1*, and *OXCT1*, were significantly downregulated in AML (**Figure 1A**). We also analyzed the transcriptional levels of the above 8 genes using mRNA expression data downloaded from GSE24006 database, including 7 AML blasts and 7 normal HSCs, and GSE65409 database including 30 AML blasts and 8 normal HSCs. The significant downregulation of 3 of the 8 genes, i.e., *BDH1*, *HADHB* and *OXCT1*, in AML, was further confirmed as compared to the normal HSCs (**Figures 1B** and **S1A, B**). This suggests the dysregulation in expression of ketone body metabolism associated genes in AML.

**Table 1:** Gene list of “Synthesis and degradation of ketone bodies”.

ENTREZID	KEGG ID	Description	Gene Symbol
3155	hsa00072	Synthesis and degradation of ketone bodies	HMGCL
3157	hsa00072	Synthesis and degradation of ketone bodies	HMGCS1
3158	hsa00072	Synthesis and degradation of ketone bodies	HMGCS2
38	hsa00072	Synthesis and degradation of ketone bodies	ACAT1
39	hsa00072	Synthesis and degradation of ketone bodies	ACAT2
5019	hsa00072	Synthesis and degradation of ketone bodies	OXCT1
54511	hsa00072	Synthesis and degradation of ketone bodies	HMGCLL1
56898	hsa00072	Synthesis and degradation of ketone bodies	BDH2
622	hsa00072	Synthesis and degradation of ketone bodies	BDH1
64064	hsa00072	Synthesis and degradation of ketone bodies	OXCT2

To further verify the findings, we performed qRT-PCR, and tested the expression levels of *BDH1*, *HADHB* and *OXCT1* in several AML cell lines, i.e., THP1/t(9;11), U937/t(10;11), Kasumi-1/t(8;21), MV4;11/t(4;11), HL-60/del(16), K562/t(9;22) (CML) and normal HSCs. The expression level of *BDH1* was significantly lower in AML cells than in normal HSCs (**Figure 1C**), in consistence with results of the above data analysis (**Figures 1A, B**). Given the fact that *BDH1* was the most broadly and consistently suppressed gene among the 3 genes investigated in AML, we focused on *BDH1* in the following study.

### *BDH1* Has a Predictive Power as a Diagnostic Biomarker for AML

**Figure 2:**
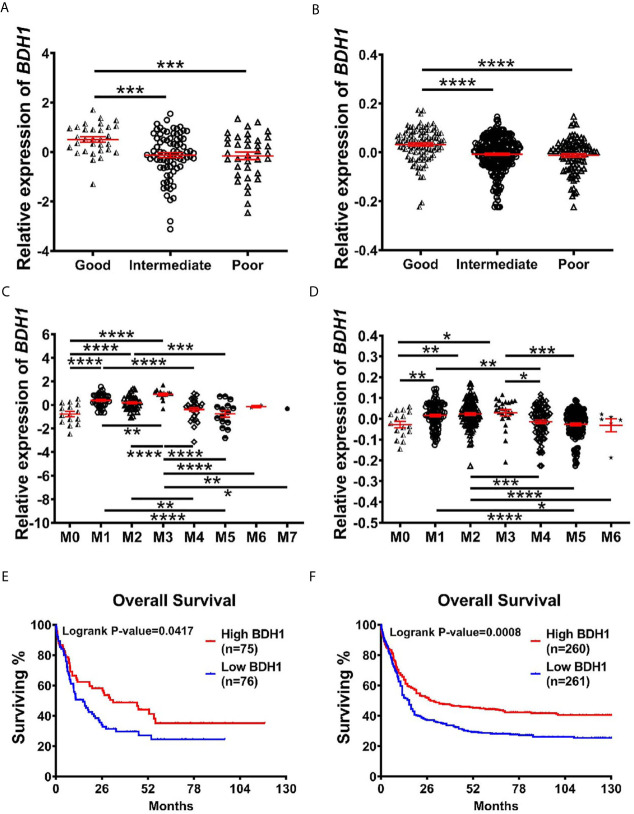
The lower expression of *BDH1* predicts the worse prognosis for AML patients. **(A, B)** Shown are comparisons of *BDH1* gene expression levels among AML patients of different cytogenetic-risk classifications in TCGA-LAML database **(A)** and GSE6891 database **(B)**, and comparison of *BDH1* gene expression among AML patients of different FAB subtypes in TCGA-LAML database **(C)** and GSE6891 database **(D)**. The expression values were log2-transformed and mean centered. The *P* values were detected by *t*-test. **(E, F)** Kaplan-Meier survival curves of *BDH1* in TCGA-LAML (left) **(E)** and GSE6891 (right) **(F)** database. *p < 0.05, **p < 0.01, ***p < 0.001, ****p < 0.0001.

In order to find out the potential relationship between *BDH1* gene expression and AML prognosis, we first compared the expression patterns of *BDH1* in different subtypes of AML. Results show that the expression level of *BDH1* was significantly lower in patients with poor cytogenetics risk than in those with favorable or intermediate cytogenetics risk (**Figures 2A, B**). The above pattern was further confirmed in another analysis according to the ELN2017 risk classification (19) (**Figure S2A**). More, AML-M3, which is classified as acute promyelocytic leukemia (APL) and has a favorable prognosis (20), was the FAB subtype that had significantly up-regulated *BDH1* expression (**Figures 2C, D** and **S2B**). Increased age is often associated with increased risk of chemoresistance and death in AML patients (21). We found that *BDH1* expression was remarkably lower in patients over 65-years old (**Figure S2C**). *CEBPA* biallelic mutations were shown to be associated with a better prognosis in AML (22). Here we found that the *BDH1* expression level in patients with *CEBPA* biallelic mutations was significantly higher than in the those with no *CEBPA* mutations (**Figures S2D, E**). The above results indicate the association between *BDH1* expression and AML clinicopathological characteristics.

Further, we investigated the potentially prognostic significance of *BDH1* expression levels in AML. Kaplan-Meier analysis of TCGA-LAML database consisting of 151 AML cases including 63 normal karyotypes, 10 inv(16), 7 t(8;21), 14 t(15;17), 7 t(11q23) and 50 other karyotypes showed that the lower expression of *BDH1* correlated with the worse overall survival (OS, log-rank test, *p*=0.0417) in AML patients (**Figure 2E**). A similar pattern was then confirmed in the GSE6891 database consisting of 521 AML cases including 187 normal karyotypes, 33 inv(16), 35 t(8;21), 21 t(15;17), 10 t(11q23) and 235 other karyotypes (OS, log-rank test, *p*=0.0008) (**Figure 2F**). These results suggest *BDH1* predicts favorable outcome in AML.

### The Level of *BDH1* Is Significantly Correlated With *HOXA9*, *MEIS1*, and *TP53* Expression Levels

**Figure 3:**
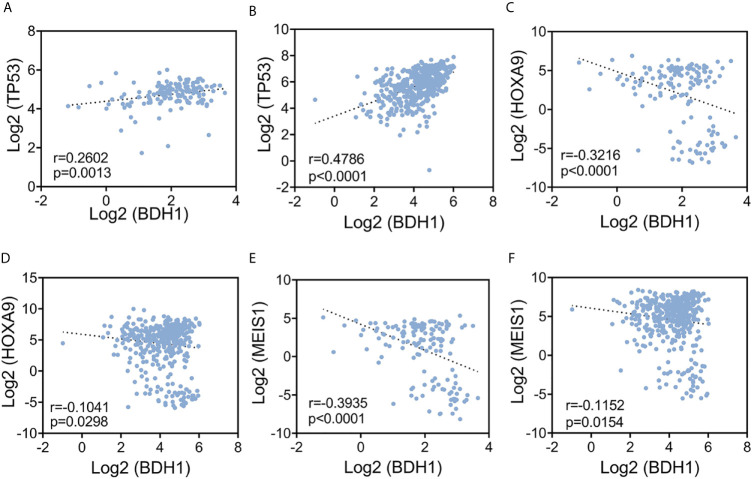
The expression level of *BDH1* is significantly correlated with that of *HOXA9*, *MEIS1*, and *TP53* in AML. Results of the pair-wise inter-correlation analysis of gene expression levels of *BDH1* and *TP53*, *HOXA9*, *MEIS1* based on TCGA-LAML database **(A, C, E)** and BEATAML database **(B, D, F)** are shown.

A variety of genes, such as *HOXA9, MEIS1* and *TP53*, have been shown to be critically involved in AML pathogenesis (23–26). The upregulation of *HOXA9* was reported in more than 50% of AML and is highly associated with poor prognosis (23). *MEIS1*, a critical regulator of leukemia stem cells (LSCs), is often found overexpressed in *Hox*-gene-driven leukemia (24). The combinational overexpression of *Hoxa9* and *Meis1* leads to a massive acceleration of leukemia development (25). As a tumor suppressor gene, *TP53* inactivation by gene deletion or mutation potently promotes AML (26). Here we investigated the correlation between expression levels of *BDH1* and *HOXA9*, *MEIS1*, or *TP53* genes in AML in TCGA-LAML and BEATAML database. Results show that the expression of *BDH1* positively correlated with *TP53* (r=0.2602, *p*=0.0013 in TCGA, r=0.4786, *p*<0.0001 in BEATAML) (**Figures 3A, B**), while negatively correlated with *HOXA9* (r=-0.3216, *p*<0.0001 in TCGA; r=-0.1041, *p*=0.0298 in BEATAML) (**Figures 3C, D**) and *MEIS1* (r=-0.3935, *p*<0.0001 in TCGA; r=-0.1152, *p*=0.0154 in BEATAML) (**Figures 3E, F**). The above results suggest the potential correlation between *BDH1* and other oncogenes and/or tumor suppressors in AML.

### *BDH1* Suppresses Cell Proliferation in *MLL-AF9* AML

**Figure 4:**
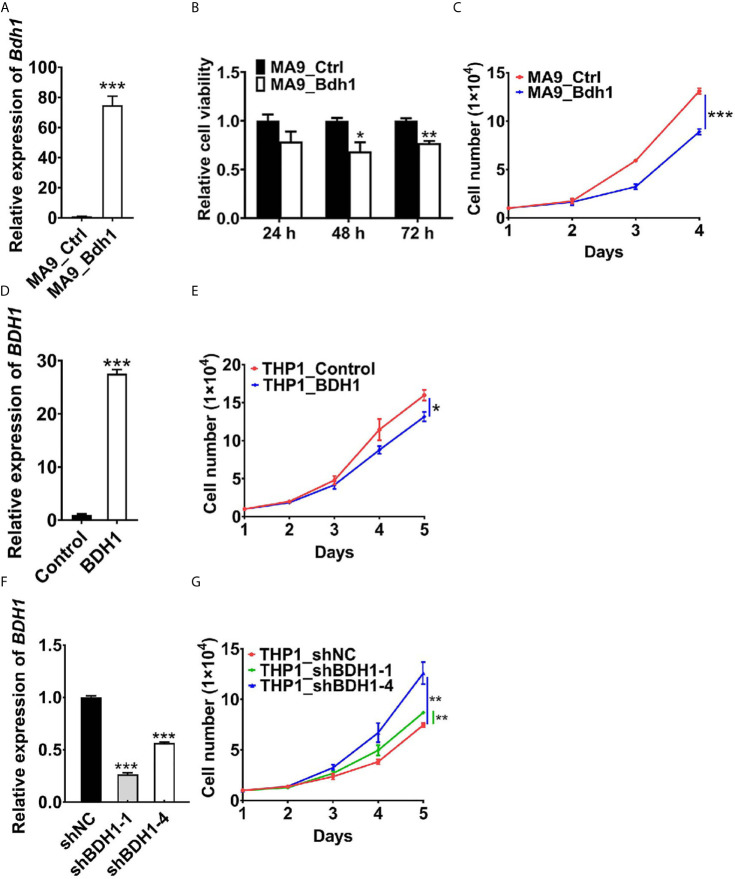
*BDH1* suppresses the growth of *MLL-AF9* AML cells. **(A)** The expression level of *Bdh1* in mouse BM cells infected with MSCVneo-MLL-AF9 (MA9), MSCV-PIG-*Bdh1* (Bdh1) or MSCV-PIG (Ctrl) retrovirus was verified through qRT-PCR after positive screening for 5 days. **(B)** *Bdh1* overexpression repressed cell viability of *MLL-AF9*-transduced mouse BM cells. Mouse BM cells were infected with MSCVneo-MLL-AF9 (MA9), MSCV-PIG-*Bdh1* (Bdh1) or MSCV-PIG (Ctrl) retrovirus. MTT assays were conducted 24, 48, 72 hours after seeding. **(C)** Cell number of each of the above groups was counted once per day until 4 days post-seeding. **(D)** The gene expression level of *BDH1* in THP1 cells infected with PLJM1-GFP (Control), PLJM1-BDH1 (BDH1) lentivirus was verified through qRT-PCR after drug screening for 7 days. **(E)** *BDH1* overexpression inhibited cell growth of THP1 cells. Cell number of each of the above groups was counted once per day until 5 days post-seeding. **(F)** The expression level of *BDH1* in THP1 cells infected with PLKO.1-TRC (shNC), PLKO.1-BDH1-shRNA-1 (shBDH1-1) and PLKO.1-BDH1-shRNA-4 (shBDH1-4) lentivirus was determined by qRT-PCR after positive screening for 7 days. **(G)** *BDH1* knock-down promoted the growth of THP1 cells. Cell number of each of the above groups was counted once per day until 5 days post-seeding. *p < 0.05, **p < 0.01, ***p < 0.001.

To assess the function of *BDH1* gene in AML, we first cloned the *Bdh1* CDS into MSCV-PIG retroviral vector, and then co-infected mouse bone marrow (BM) progenitor cells with MSCVneo-*MLL-AF9* (MA9) and MSCV-PIG-*Bdh1* (Bdh1) or MSCV-PIG (Ctrl) retroviruses. The expression of exotic *Bdh1* was confirmed at mRNA level with real-time RT-PCR assays (**Figure 4A**). We found that cell viability of MA9 BM cells overexpressing *Bdh1* was significantly lower than control group (**Figure 4B**). *Bdh1* overexpression remarkably suppressed cell proliferation (**Figure 4C**). In order to interfere *BDH1* expression in human AML cell line, human *BDH1* was cloned into PLJM1-EGFP vector, and PLJM1-EGFP was used as the negative control. Then THP1/t(9;11) cells were infected with PLJM1-EGFP (Control) or PLJM1-BDH1 (BDH1) lentivirus. The expression of exotic *BDH1* was confirmed at mRNA level with real-time RT-PCR assays (**Figure 4D**). Forced expression of *BDH1* significantly inhibited cell proliferation in THP1 cells (**Figure 4E**). We then knocked down *BDH1* expression by use of shRNAs. In contrast, the speed of proliferation of THP1 cells with *BDH1* knockdown was significantly higher than that of the control cells (**Figures 4F, G**). Taken together, our data verified that *BDH1* exhibited an anti-tumor effect in MA9 AML cells.

## Discussion

Despite of dysregulation of the aerobic glycolysis (3–5), the amino acid (6) and fatty acid metabolism (7, 8), etc., the role and regulatory mechanism of ketone metabolism in AML still remain obscure. In this study, through analysis of several AML databases, we first showed the downregulation of *BDH1*, a key regulator of ketone body metabolism in AML blasts, as compared with normal HSCs. Then we proved that *BDH1* expression positively correlated with AML prognosis. Through *in vitro* studies in human and mouse AML cells, we further confirmed the anti-tumor effect of *BDH1* in AML. Our findings not only reveal the previously unappreciated anti-tumor function of *BDH1* in AML, but also indicate the potential of targeting *BDH1* to cure AML.

Abnormal metabolism such as enhanced glycolysis, nutrient addiction, lipid metabolism dysregulation, has been shown to be involved in AML pathogenesis (27). Therefore, targeting metabolic pathways holds great potential in treating AML. For example, the Internal tandem duplication (ITD) mutations in Fms-like tyrosine kinase 3 gene (*FLT3*-ITD) are known to be associated with the upregulation of the glycolytic gate keeper enzyme hexokinase 2 (HK-2) and increased glycolytic activity in AML. Glycolytic inhibitors such as 3-Bromopyruvate propyl ester (3-BrOP) and 2-deoxyglucose (2-DG) exhibited cytotoxicity in murine and human leukemia cells carrying FLT3/ITD mutation (28). Lise et al. found that knockdown of *SLC1A5*, the transporter for glutamine uptake, could induce apoptosis in MOLM-14 AML cells and inhibit tumor formation in the mouse AML xenotransplantation model (6). More, Yoko et al. showed that inhibition of fatty acid oxidation (FAO) by Etomoxir (FAO inhibitor) induced apoptosis in U937 AML cells co-cultured with BM adipocytes (29). And it was recently reported that BRQ (dihydroorotate dehydrogenase (DHODH) inhibitor) treatment could sensitize chemoresistant AML cells in AML mice (30). However, knowledge of some metabolic pathways, such as the ketone metabolism, in cancer, and especially in AML, still remains limited. Therefore, it is crucial to further explore into various metabolic pathways in AML, and develop novel targeted therapy based on a better understanding of the molecular mechanisms of each metabolic pathway.

Among these metabolisms, ketones are known as an important alternative energy source during metabolic stress. The KD is regarded as a promising adjuvant and a patient-specific multifactorial cancer therapy. As a key regulator in both ketogenesis and ketolysis, *BDH1* has been shown to determine the cell fate of adipocytes (31, 32). However, the role of *BDH1* in cancer still remains unclear. The expression levels of *BDH1* vary among different cancer cells. Low expression levels of ketolytic enzymes, including BDH1, have been found in tumors such as pancreatic cancer and gliomas (12, 33, 34). *BDH1* expression level is closely associated with the amount of ketone body in cancer cell lines (35). βOHB treatment was shown to be able to partly rescue the growth inhibition effect of low glucose in Hela cells, which has relatively high *BDH1* expression, but not in PNAC-1 cells with low *BDH1* level (33). The role of *BDH1* and ketone body in cancer and cancer therapy still remains quite controversial. Here we show the anti-tumor role of *BDH1* in AML, which enlightens the possibility of restoration of *BDH1* expression as an AML therapy. It is highly possible that KD would benefit AML patients especially those with abnormal *BDH1* expression and dysregulated ketone metabolism.

In our present work, we found that the transcriptional inactivation of *BDH1* significantly correlated with poor prognosis and shorter survival in AML patients. Thus, the gene expression level of *BDH1* has a great power in AML diagnosis and prognosis. We further confirmed that *BDH1* expression level was negatively correlated with the expression levels of oncogenes, e.g., *HOXA9* and *MEIS1*, but was positively correlated with the tumor suppressor *TP53*. The regulatory mechanisms of *BDH1* on AML leukemogenesis and therapy, as well as on the expression of *HOXA9, MEIS1* and *TP53* are no doubt worth further investigation.

## Conclusions

From the present research, we show the downregulation and the anti-tumor effects of *BDH1* in AML. The expression level of *BDH1* is positively correlated with AML prognosis. Our study not only provides novel insights into the mechanism of AML pathogenesis and metabolism, but also indicates the potential of targeting *BDH1* and ketone metabolism in treating AML.

## Data Availability Statement

The raw data supporting the conclusions of this article will be made available by the authors, without undue reservation.

## Ethics Statement

The studies involving human participants were reviewed and approved by the institutional ethics review board of the First Affiliated Hospital of Zhejiang University and Zhejiang University School of Medicine. The patients/participants provided their written informed consent to participate in this study. The animal study was reviewed and approved by the ethics committee of the Laboratory Animal Center of Zhejiang University.

## Author Contributions

FH and XJ conceived the study and wrote the paper. FH, HZ, JL, WY, LY, YL, DS, XC, SZ, HJ, XL, JS, HH, QW, and XJ performed the experiments and data analysis. All authors contributed to the article and approved the submitted version.

## Funding

This work was supported by the National Natural Science Foundation of China (Grant Nos. 31900426 and 81970144) (to XJ).

## Conflict of Interest

The authors declare that the research was conducted in the absence of any commercial or financial relationships that could be construed as a potential conflict of interest.

## Abbreviations

AML, acute myeloid leukemia; AcAc, acetoacetate; βOHB, βhydroxybutyrate; AcAc-CoA, acetoactyl-CoA; HMGCS2, 3-hydroxymethylglutaryl-CoA synthase; HMG-CoA, hydroxymethylglutaryl-CoA; HMGCL, Hydroxymethylglutaryl coenzyme A lyase; KD, ketone diet; IL-3, interleukin 3; 5-FU, 5-fluorouracil; qRT-PCR, quantitative real-time PCR; GEO, Gene Expression Omnibus; KEGG, Kyoto Encyclopedia of Genes and Genomes; HSCs, hematopoietic stem cells; APL, acute promyelocytic leukemia; OS, overall survival; BM, bone marrow; ITD, Internal tandem duplication; FLT3, Fms-like tyrosine kinase 3; HK2, hexokinase 2; 3-BrOP, 3-Bromopyruvate propyl ester; 2-DG, 2-deoxyglucose; FAO, fatty acid oxidation; DHODH, dihydroorotate dehydrogenase.
