# Supplementary figures and images for: Anti-Tumor Effects of BDH1 in Acute Myeloid Leukemia
Source: Front Oncol. 2021 Jun 4;11:694594. doi: 10.3389/fonc.2021.694594 (PMC8213090; doi:10.3389/fonc.2021.694594)

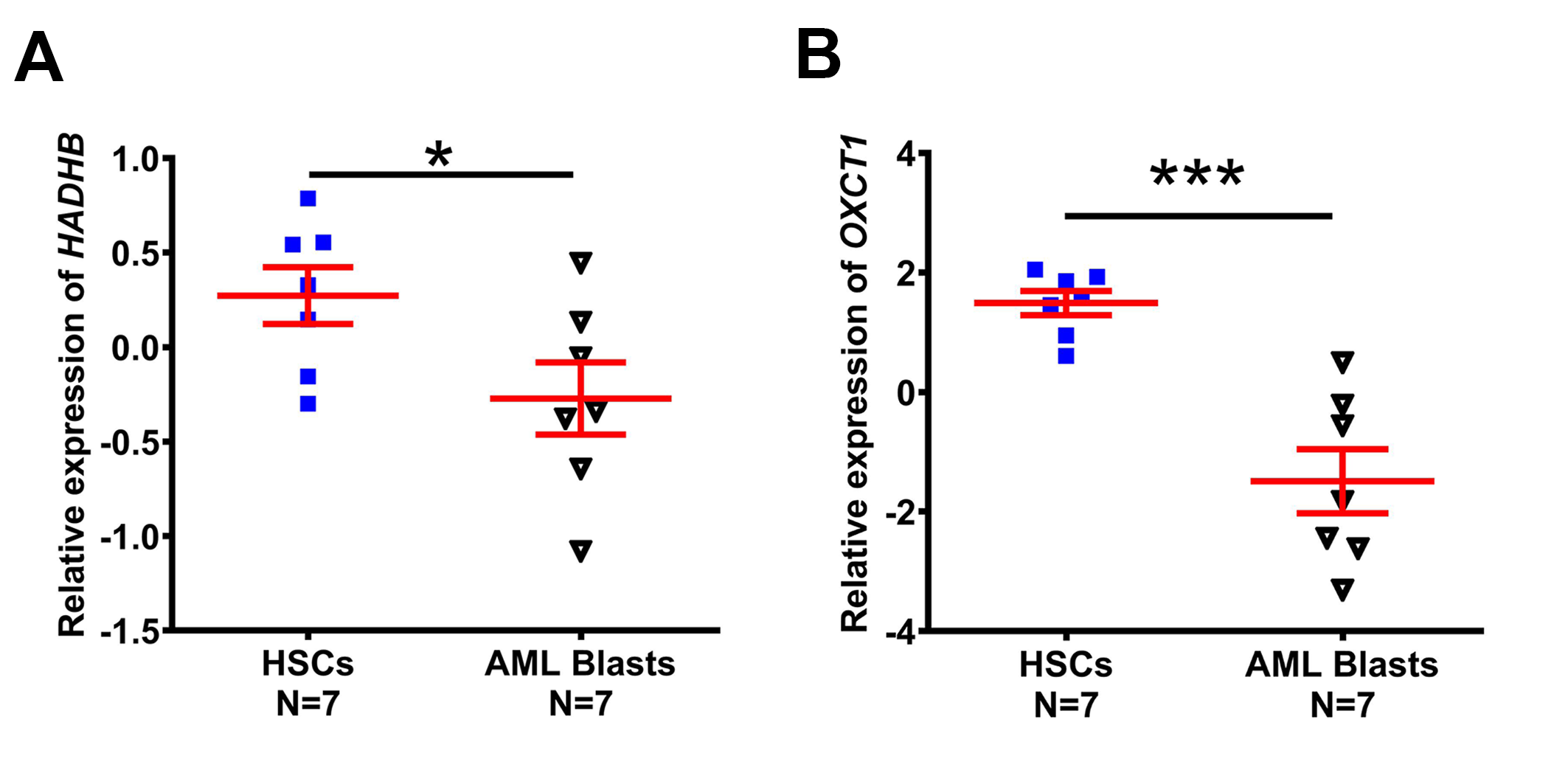

Supplement: Supplementary file 1 [file Image_1.tif]

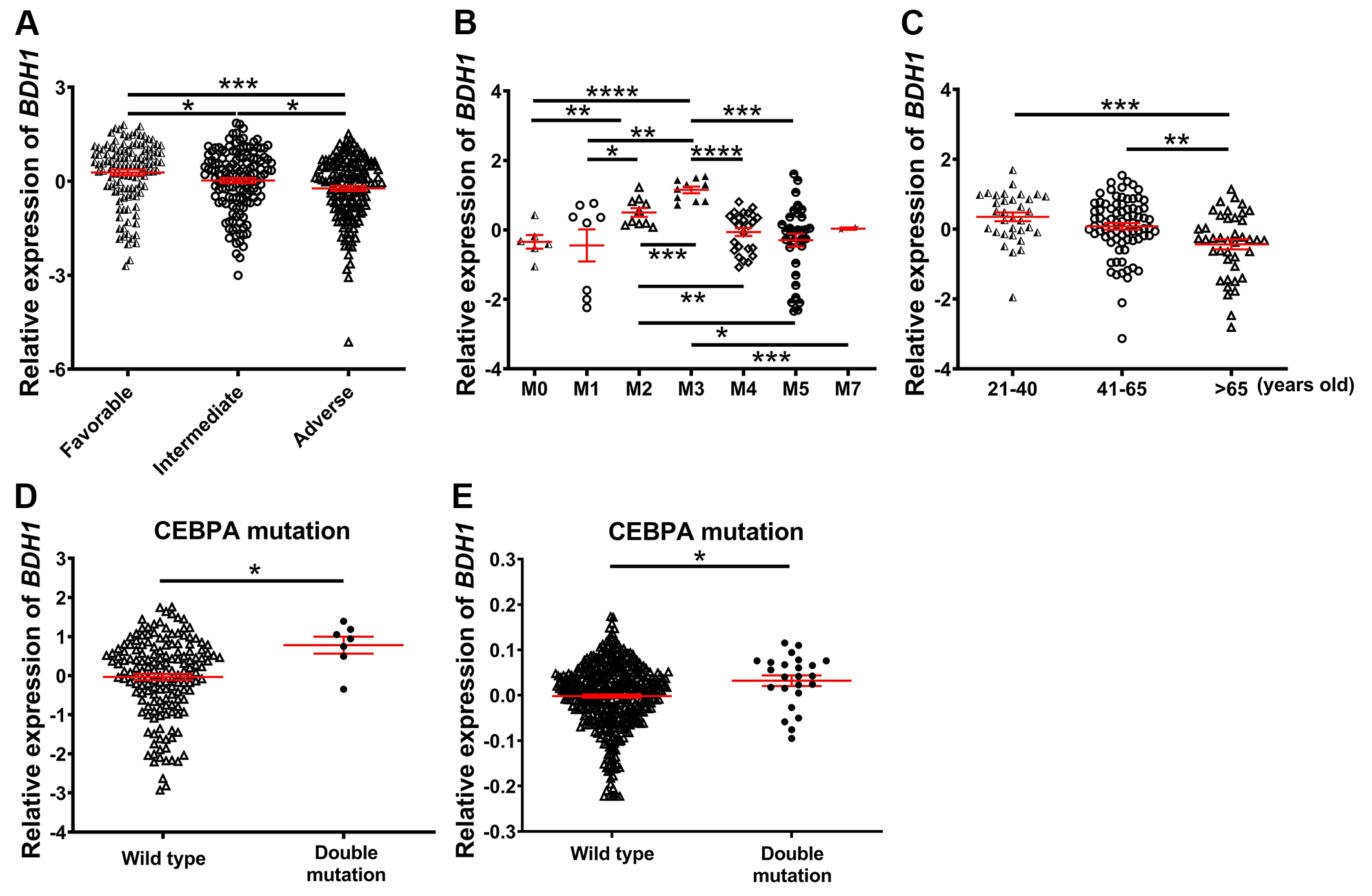

Supplement: Supplementary file 2 [file Image_2.tif]
